# Bridging the Gap: Biofilm-mediated establishment of *Bacillus velezensis* on *Trichoderma guizhouense* mycelia

**DOI:** 10.1016/j.bioflm.2024.100239

**Published:** 2024-11-16

**Authors:** Jiyu Xie, Xinli Sun, Yanwei Xia, Lili Tao, Taimeng Tan, Nan Zhang, Weibing Xun, Ruifu Zhang, Ákos T. Kovács, Zhihui Xu, Qirong Shen

**Affiliations:** a Jiangsu Provincial Key Lab for Solid Organic Waste Utilization, Key Lab of Organic-based Fertilizers of China, Jiangsu Collaborative Innovation Center for Solid Organic Wastes, Educational Ministry Engineering Center of Resource-saving Fertilizers, Nanjing Agricultural University, Nanjing, 210095, China; b Institute of Biology Leiden, Leiden University, 2333 BE, Leiden, the Netherlands

**Keywords:** *Bacillus velezensis*, *Trichoderma guizhouense*, Biofilm, Hyphae colonization, EPS, TasA

## Abstract

Bacterial-fungal interactions (BFIs) are important in ecosystem dynamics, especially within the soil rhizosphere. The bacterium *Bacillus velezensis* SQR9 and the fungus *Trichoderma guizhouense* NJAU 4742 have gathered considerable attention due to their roles in promoting plant growth and protecting their host against pathogens. In this study, we utilized these two model microorganisms to investigate BFIs. We firstly demonstrate that while co-inoculation of *B. velezensis* and *T. guizhouense* could promote tomato growth, these two microorganisms display mutual antagonism on agar solidified medium. To resolve this contradiction, we developed an inoculation method, that allows *B. velezensis* colonization of *T*. *guizhouense* hyphae and performed a transcriptome analysis. During colonization of the fungal hyphae, *B. velezensis* SQR9 upregulates expression of biofilm related genes (e.g. *eps, tasA*, and *bslA)* that is distinct from free-living cells. This result suggested an intricate association between extracellular matrix expression and hyphae colonization. In accordance, deletion *epsD*, *tasA, or* both *epsD* and *tasA* genes of *B. velezensis* diminished colonization of the *T*. *guizhouense* hyphae. The insights from our study demonstrate that soil BFIs are more complex than we understood, potentially involving both competition and cooperation. These intricate biofilm-mediated BFI dynamics might contribute to the remarkable diversity observed within soil microbiota, providing a fresh perspective for further exploration of BFIs in the plant rhizosphere.

## 1 Introduction

In nearly all ecosystems, bacteria and fungi frequently assemble into dynamic co-evolving communities, encompassing microbial species from a wide diversity of fungal and bacterial families [1]. These bacterial-fungal interactions (BFIs) are vital in ecosystems, driving biochemical cycles and impacting the health of plants and animals [2,3]. The complex interplay between bacteria and fungi not only shapes natural environments but also has significant implications for human activities. As a result, understanding and harnessing these BFIs led to diverse applications in agriculture, forestry, environmental protection, food processing, biotechnology, and medicine, offering solutions to various challenges and fostering innovation [4]. Generally, physical association between bacteria and fungi includes planktonic co-cultures, mixed biofilms, and intrahyphal colonization [5]. Here, we concentrate on BFI through biofilm formation. Biofilms are the collective lifestyle of most microorganisms on earth, where cells are embedded within a self-produced extracellular polymeric substances adhering to each other and surface [6]. These complex structures play a crucial role in BFIs by providing a platform for close proximity interactions. The matrix contributes to the structure of a biofilm, allowing for the exchange of metabolites, signaling molecules, genetic material, and defensive compounds between different microbial species [7,8]. This intimate association within biofilms facilitates the formation of dynamic microbial communities and underlies many of the ecological function of BFIs. However, research on BFIs within biofilms is currently limited due to significant differences in growth rates, complex patterns, and other factors between bacteria and fungi.

The pattern of bacterial and fungal abundances within biofilm communities arise from intimate biophysical and metabolic interactions, leading mutual dependency and co-evolution between these microbial partners [9]. This co-evolution is evident in various ecosystems. For instance, in soil, *Pseudomonas fluorescens* BBc6R8 enhances the survival of the mycorrhizal fungus *Laccaria bicolor* in challenging soil conditions [10], and *L. bicolor* also supports the survival of the bacterium [11]. BFIs can also facilitate the colonization of surfaces that would otherwise be inaccessible to certain microorganisms. Using an *in vitro* polystyrene-serum system, *Candida albicans* was shown to strongly enhance biofilm formation of *Staphylococcus aureus*, where the bacterial cells associated with the fungal hyphae rather than the plastic substrate [12].

Among the various BFIs occurring in biofilms, those involving plant growth promoting microorganisms (PGPMs) are of particular interest due to their significant impact on agriculture. *Trichoderma* and *Bacillus* as the most extensively studied PGPMs, with research focusing on elucidating the mechanisms for plant growth promotion [13,14]. *Trichoderma guizhouense*, an opportunistic and non-pathogenic plant symbiotic fungus, contributes to plant health by directly inhibits plant pathogens through parasitism [15] and indirectly by inducing local and systemic defenses in plants [16], ultimately leading to enhanced root development and plant growth. Similarly, *Bacillus velezensis*, a Gram-positive soil-dwelling bacterium, is non-pathogenic and commonly resides in association with the plant rhizosphere [17]. Importantly, *Bacilli* rely on their ability to form biofilms to exert their beneficial effects, highlighting the significance of biofilm formation in these interactions. *B. velezensis* and members of the *B. subtilis* species complex have been reported to develop biofilms on various abiotic and biotic surfaces that depends on the production of biofilm matrix components [[18], [19], [20], [21]]. Several studies have reported that the co-inoculation of *Trichoderma* sp. and *Bacillus* sp. into the rhizosphere enhance their plant-growth-promoting abilities and disease suppression [22,23]. However, the underlying mechanisms of this synergistic interaction, particularly within the context of biofilm formation, have not been thoroughly investigated.

Building upon the existing knowledge of BFIs and the importance of biofilms in PGPMs activities, our study aimed to elucidate the interactions observed between a bacterium and a fungus with plant growth promotion ability. Specifically, we investigate the interaction between the fungus *T. guizhouense* NJAU 4742 and bacterium *B. velezensis* SQR9, both of which have been reported to exhibit a synergistic growth-promotion on tomato plants when co-inoculated [24]. To overcome the challenge of growth rate disparities often encountered in BFIs research, we established a cultivation method designed to mitigate this drawback. Our approach combines transcriptome analysis, construction of biofilm mutants, and microscopy approaches including stereo microscopy, confocal laser scanning microscopy (CLSM), and scanning electron microscopy (SEM). Transcriptome analysis suggested that hyphae-colonized *B*. *velezensis* display a distinct gene expression profile compared with non-attached cells and bacterial mono-culture samples, with notable up regulation of biofilm formation-related genes. Furthermore, we demonstrate that biofilm formation by *B. velezensis* is essential for its colonization of *T. guizhouense* hyphae, which we hypothesize that it might facilitate a synergistic interaction. This study not only advances our understanding of the molecular basis of BFIs but also offers suggestions on potential strategies for enhancing the efficacy of microbial inoculants in agriculture.

## 2 Material and methods

### 2.1 Strains and growth conditions

The bacterial and fungal strains in this study are listed in Table 1. *B. velezensis* strain SQR9 was grown at 37 °C in Lysogeny Broth medium (LB-Lennox, Carl Roth, Germany; tryptone10 g/L, yeast extract 5 g/L and NaCl 5 g/L) supplemented with 1.5 % Bacto agar if required. *T. guizhouense* strain NJAU 4742 was grown at 28 °C in Potato Dextrose Agar (PDA, BD Difco, USA) medium for 7 days with light. Spores were harvested with 5 ml water and filtered through Miracloth. Spores were stored at 4 °C until use. When required, antibiotics were added to media at the following final concentrations: chloramphenicol (*Cm*) at 5 μg/mL, erythromycin (Em) at 1 μg/mL, spectinomycin (Spec) at 100 μg/mL and Hygromycin B (Hyg) at 100 μg/mL.

**Table 1:** Bacterial and fungal strains used in this study.

Strains	Genotype	Reference
*B. velezensis* SQR9	Wild type	[25]
gfp labeled *B. velezensis* SQR9	wild type with pNW33n-gfp, Cm^R^	[26]
gfp labeled *B. velezensis* Δ*epsD*	Δ*epsD*::Spec^R^, pNW33n-gfp Cm^R^	This study
gfp labeled *B. velezensis* Δ*tasA*	Δ*tasA*::Em^R^, pNW33n-gfp Cm^R^	This study
gfp labeled *B. velezensis* Δ*epsD*Δ*tasA*	Δ*epsD*::Spec^R^ Δ*tasA*::Em^R^, pNW33n-gfp Cm^R^	This study
*T. guizhouense* NJAU 4742	Wild type	[15]
*T. guizhouense* NJAU 4742-mcherry	*T. guizhouense* NJAU 4742 *mCherry,* Hyg^R^	[27]

Cm^R^, Em^R^, Spec^R^ and Hyg^R^ denote chloramphenicol, erythromycin, spectinomycin and Hygromycin B resistance markers.

### 2.2 Bacteria-fungi coculture assay

**Fig. 1:**
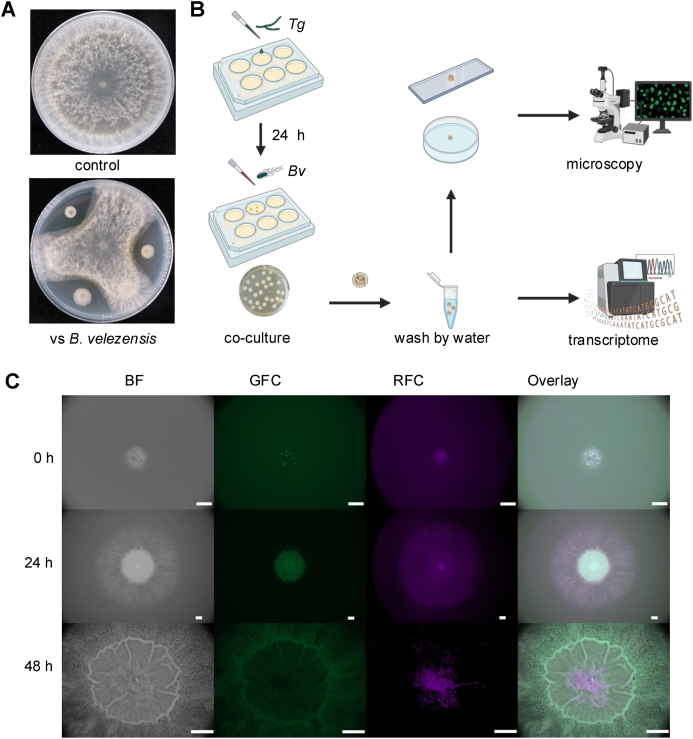
**Colonization of the *T. guizhouense* hyphae by *B. velezensis***. (A) The interaction between *T. guizhouense* and *B. velezensis* on agar plates. The diameter of Petri dish plate: 9 cm. (B) The experiments setup and analysis method*. T. guizhouense* was cultured for 24h, followed by co-culture with *B. velezensis*. Hyphal pellets were washed three times by water. Samples were used for transcriptome analysis and microscopic observations. (C) Co-culture images of *B. velezensis* (carrying GFP) and *T. guizhouense* (carrying mCherry) by stereo microscopy. Images were taken in 0h, 24h, 48h. BF: bright channel; GFC: green fluorescence channel; RFC: red fluorescence channel. Scale bars indicate 1000 μm.

**Fig. 2:**
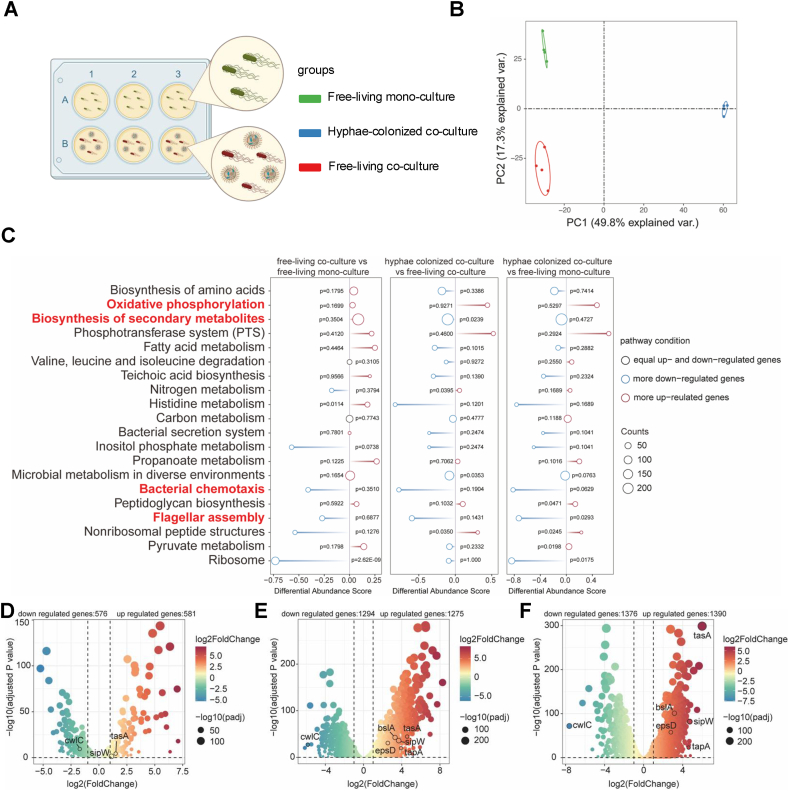
**Transcriptome analysis of *B. velezensis***. (A) Schematic representation of transcriptome treatments. (B) Principal Coordinates Analysis (PCoA) of *B. velezensis* is plotted based on the Bray−Curtis distance metrices for taxonomical data (p < 0.01). Permutational multivariate analysis of variance (PERMANOVA) was performed using the adonis function from the vegan R package. Samples were collected from different treatment (n = 4). (C) KEGG enriched pathway analysis of all three treatments. Blue indicated there were more down-regulated genes in the pathway, red indicated there were more up-regulated genes in the pathway, black indicated there were equal up-and-down regulated genes in the pathway. (D) The log2 (fold change) of genes in the free living cocultured *B. velezensis* compared with mono-culture. (E) The log2 (fold change) of genes in the hypha colonized *B. velezensis* compared free-living co-culture. (F) The log2 (fold change) of genes in the hyphae colonized *B. velezensis* compared mono-culture. Numbers of the differentially regulated genes in three treatments were showed on the top of figures. LFC >2, *p* < 0.05, *t*-test.

4 μL spores (10^8^ spores/mL) of *T. guizhouense* were inoculated into a single well of 6 well plates with 4 ml LB medium and incubated at 28 °C with shaking at 180 rpm. After 24h, 4 μL B*. velezensis* culture with an optical density of 1.0 at 600 nm (OD_600_ = 1.0) was inoculated into each well. The plates were incubated at 28 °C for 24h with shaking at 180 rpm. After incubation, the fungal pellets were transferred to new tubes and washed three times with distilled water (Fig. 1B). Bacterial cells that still remained colonized on the hyphae after washing were termed “hyphae colonized”, while those present in the co-culture medium were termed “free-living coculture” (Fig. 2A). The samples were used for Microscopy observation or RNA extraction.

### 2.3 Plant experimental design

The schematic diagram of the plant experiment is shown in Fig. S1. The tomato plant experiment was conducted in the greenhouse of Nanjing Agricultural University. Soil for plant experiments was sourced from Nanjing, Jiangsu Province, China. Tomato seeds were surface-disinfected in 2 % sodium hypochlorite for 3 min, rinsed three times in sterile distilled water and germinated in 9 cm Petri dishes covered with sterile wet filter paper at 28 °C. One-week-old tomato seedlings were transferred into the pots containing 2 kg soil and grown for another week. Six treatments were then desined as follows: CTL(control); CF(chemical fertilizer, N input 0.15 g/kg) [28]; Bv (B. velezensis alone with chemical fertilizer); Tg (*T. guizhouense* alone, with chemical fertilizer); Bv + Tg (inculated *B. velezensis* in the soil first, then inoculated *T. guizhouense* after one-week growth, with chemical fertilizer); Tg + Bv (inoculated *T. guizhouense* in the soil first, then inoculated *B. velezensis* after one-week growth, with chemical fertilizer). Strains were mixed into the soil at concentration of 10^7^ spores/g soil for *T. guizhouense* and 10^8^ CFU/g soil for *B. velezensis*. Plants were grown at 30 °C with a 16h light/8h dark cycle. Each treatment included three replicates.

### 2.4 RNA-seq analysis

Samples preparation: after 24h of co-cultivation (Fig. 1B), the hyphal pellets were washed three times by water. The suspended cell from this washing process were used as the free-living coculture treatment, samples were collected by centrifuge. The cells that attached to the hyphal pellets after washing were considered as the hyphae-colonized treatment (Fig. 2A).

Total RNA was extracted using the E. Z.N.A. bacterial RNA kit (Omega Bio-tek, Inc.). RNA integrity was assessed using the RNA Nano 6000 Assay Kit of the Bioanalyzer 2100 system (Agilent Technologies, CA, USA). mRNA was isolated from total RNA and fragmented. cDNA synthesis was performed using *M*-MuLV Reverse Transcriptase and DNA polymerase I, with dUTP in the second strand. After adapter ligation, the second strand was degraded by USER Enzyme. Size selection was done using AMPure XP system (Beckman Coulter, Beverly, USA), followed by PCR with Phusion High-Fidelity DNA polymerase and library quality assessment on Agilent Bioanalyzer 2100 system, and subsequently sequenced on TruSeq PE Cluster Kit v3-cBot-HS (Illumina) platform. Raw reads were quality-trimmed and then mapped to reference genomes using Bowtie2-2.2.3 software [29]. HTSeq v0.6.1 was used to count the reads numbers mapped to each gene. And then FPKM of each gene was calculated based on the length of the gene and reads count mapped to this gene. Differential expression analysis was performed using the DESeq2 R package [30] The resulting p values of genes were adjusted using Benjamini and Hochberg's approach for controlling the false discovery rate (FDR). Genes were assigned as differentially expressed when log2 fold change (LFC) > 2 and FDR <0.05. For functional analysis, the protein-coding sequences were mapped with KEGG Orthology terms using EggNOG-mapper v2 [31]. P values of pathways were corrected for multiple hypothesis testing using the Benjamini and Hochberg's approach.

### 2.5 Microscopy

A confocal laser scanning microscope Axio Observer Z1/7 (Carl Zeiss) equipped with a 20 × /0.50 M27 EC Plan-Neofluar objective, was used to acquire confocal microscopic images. *B. velezensis* was excited with 488 nm laser and detected at 509 nm for GFP signal. A stereo-microscope Axio Zoom V17 (Carl Zeiss) equipped with a 1.0 × Plan-Neofluar objective, was used to acquire images of plates (with different magnifications). Fluorescence of *B. velezensi*s and *T. guizhouense* samples were detected at 488 nm(Ex) and 409 nm (Em) and 587 nm (Ex) and 610 nm (Em) for the GFP and mCherry signals, respectively. Images were collected with Zen software (Carl Zeiss) and analyzed using the ImageJ software. For scanning electron microscopy, the samples were fixed with 2.5 % electron microscopy grade glutaraldehyde and 2 % osmium tetroxide (OsO) in distilled water. The sample were dried using tert-butanol and ethanol. After coating, the samples were imaged with a Scanning Electron Microscope Regulus 8100 (Hitachi High-Tech, Tokyo, Japan).

### 2.6 Construction of gene deletion bacterial strains

Deletion of *tasA* and *epsD* genes was performed using overlap-PCR based strategy as previously described [32]. The upstream (800 bp) and downstream regions (1000 bp) were amplified from wild-type *B. velezensis* genome using primer pairs (Table S1) tasA_UF/tasA_UR, epsD_UF/eps_UR and tasA_DF/tasA_DR epsD_DF/epsD_DR, respectively. The antibiotic markers (Em and Spec) were amplified from plasmids pPax01 [33] and pheS-SPC [34] using the primer pairs Em_F/Em_R and Spc_F/Spc_R, respectively. The three fragments were fused using overlay PCR in the order of upstream, antibiotic region and downstream fragments. The resulting *tasA* and *epsD* deletion amplicons (PCR products) were directly transformed into *B. velezensis* containing the constitutive GFP (*B. velezensis* SQR9-gfp), and the transformants were selected on LB agar medium containing erythromycin, spectinomycin, or both erythromycin and spectinomycin, to obtain the *tasA*, *epsD* or *tasA-epsD* deletions, respectively. Mutants were confirmed using tasA_F/tasA_R and epsD_F/epsD_R, and the PCR product was further validated by DNA sequencing.

## 3 Results

### 3.1 Co-inoculation of *B. velezensis* and *T. guizhouense* maintains their plant growth-promotion

In laboratory conditions, co-incubation of *B. velezensis* and *T. guizhouense* on PDA agar medium revealed a strong competitive interaction (Fig. 1A), attributed to the antifungal secondary metabolites produced by *B. velezensis* [35]. The observed inhibitory activity between the bacterium and fungus raises questions about their ability to co-exist when applied as plant growth promoting agents. However, sequential inoculation of these two PGPMs might mitigate their mutual inhibition, potentially allowing for their successful co-application.

To investigate the effect of subsequent co-inoculation of *B. velezensis* and *T. guizhouense* on tomato growth, we used a pot experimental setup as depicted in Fig. S1. In *Bv* + *Tg* treatment, we initially incubated two-week-old tomato plant seedlings with *B. velezensis* and incubated for one week, followed by subsequent inoculation of *T. guizhouense*. Conversely, *Tg* + *Bv* treatment involved the reverse experiments. After six weeks of cultivation, notable differences in tomato growth were recorded (Fig. S2A). The visual plant growth as well as the plant fresh weight (Fig. S2B) quantification demonstrated that treatments with PGPM significantly enhanced plant growth compared with the control and the chemical fertilizer treatments. Interestingly, despite mutual antagonism *in vitro*, *B. velezensis* and *T. guizhouense* were able to enhance tomato growth when co-inoculated to the roots of tomato plants.

### 3.2 *B. velezensis* can colonize the pre-grown hyphae of *T. guizhouense in vitro*

To elucidate the contradiction between the antagonism observed between *B. velezensis* and *T. guizhouense* on agar medium and the lack of inhibitory activity on each other, but still facilitating plant growth when inoculated subsequently, we further tested the interaction among these two species under laboratory settings. Importantly, our experiments did not aim to reproduce and explain the complex interactions that are present in the plant rhizosphere, but we aimed to test this specific BFI in a simple laboratory condition to identify molecular mechanisms occurring during their interaction. Considering the growth rate of fungi and bacteria might differ on laboratory media, we hypothesized that these two strains could achieve co-existence through controlling the time of inoculation. Here, the slower growth rate of the fungus, *T. guizhouense* might carry a disadvantage during this specific BFI. Thus, we implemented a cultivation method [20,36] aiming at alleviating the competitive pressure resulting from tentative differential growth rates (Fig. 1B). Initially, we inoculated the spores of mCherry-labeled *T. guizhouense* in a 6-well plate, allowing for spore germination during a 24-h period with shaking. After a day of incubation at 28 °C, 20–30 hyphal pellets of *T. guizhouense* were observed in each well. Subsequently, GFP-labeled *B. velezensis* was directly inoculated into the fungal cultures and the co-culture was incubated for 24 h.

To dissect whether this preincubation step facilitates interaction among the two PGPMs, the non-attached *B. velezensis* cells were first removed by washing the hyphae pellets using sterile water and BFI pellet was placed onto PDA medium. Surprisingly, we discovered that this approach allowed the simultaneous growth of both *B. velezensis* and *T. guizhouense* (Fig. 1C). Although only a small amount of green fluorescence was observed at the start, after 24 h of incubation, the presence of *B. velezensis* was evident in the center of the colony, while *T. guizhouense* spread radially outwards. This observation suggests that within such coculture setup, *T. guizhouense* and *B. velezensis* lack direct inhibition of each other. After 48 h of incubation, *B. velezensis* formed a wrinkled structure at the center whilst the hyphae of *T. guizhouense* further extended towards the non-colonized region of the agar medium. In summary, when *T. guizhouense* was pre-cultivated before co-cultivation with *B. velezensis,* an interaction could be observed that enabled *B. velezensis* to colonize the hyphae of *T. guizhouense* and continue to grow in subsequent cultivations.

### 3.3 *B. velezensis* differentially regulates biofilm and motility-related genes during colonization of the fungal hyphae

To gain a detailed insights into the hyphae colonization mechanism by the bacterium, the transcriptome of *B.* velezensis was compared using three conditions: control (bacterial monoculture), free-living bacterial cells in co-culture, and hyphae attached bacterial cells (Fig. 2A). First, principal coordinates analysis (PCoA) was employed to visualize the transcriptome differences of the bacterial cultures under these conditions using Bray-Curtis distances (Fig. 2B). The three sample types were clearly separated on the coordinates. To compare gene expression among the different treatments, we selected 20 major pathways (Fig. 2C). Compared with the free-living coculture samples, the hyphae-colonized *B. velezensis* cells exhibited downregulation of genes from 14 functional categories and upregulation of genes from 6 functional categories. Major downregulated functional categories included flagellar assembly, bacterial chemotaxis and histidine metabolism. The decreased expression of histidine metabolism suggests a reduced rate of protein synthesis and cellular growth [37]. Furthermore, the reduced expression of bacterial genes encoding flagellar assembly [38] and bacterial chemotaxis [39] indicates a decreased cell motility, consistent with the sessile colonization of the fungal hyphae. The major upregulated functional categories included phosphotransferase system. This suggests that cells were adapting to the new environment through multiple pathways, potentially involving changes in metabolism, signaling, and gene regulation. Notably, as our analysis is based on a single time point during the interaction between a bacterium and a fungus, the data provide initial hypotheses on the interaction between *B. velezensis* and *T. guizhouense*. Future time series transcriptome experiments will be required to obtain a complete understanding of BFI.

Similarly, we found reduced metabolism and motility gene expression in hyphae-colonized cells compared with free-living monoculture (Fig. 2C). Additionally, comparison of free-living cells in the coculture and the monoculture, 12 up and 6 downregulated functional categories could be detected in the cocultured *B. velezensis*. The category with the highest number of differential gene expression included the biosynthesis of secondary metabolites [40], which suggests a bacterial stress response to inhibit the fungus (Fig. 2C).

Following this, we conducted a differential gene expression analysis to reveal the significant differences in *B. velezensis* transcriptome during fungal hyphae colonization (Fig. S3). Specifically, 1275 genes were upregulated while 1294 genes were downregulated in the *B. velezensis* cells colonizing the hyphae compared with the free-living cocultures (Fig. 2E). Conversely, there were only 581 and 576 genes were up- and downregulated in the cocultured free living *B. velezensis* compared with the monocultures (Fig. 2D). Notably, the *tasA* gene, which encodes the amyloidogenic protein of the biofilm matrix in *B. velezensis* [41], was upregulated in hyphae-colonized cells compared with both monoculture and free-living coculture conditions, especially in comparison with the monoculture (Fig. 2F). Subsequently, the expression of biofilm-related genes of *B. velezensis* [17] was examined in the transcriptome, including the *eps* operon, *bslA*, *tapA* operon (that contains also the *sipW* and *tasA* genes) (Fig. 2D–F). The *epsA-O* operon encodes the synthesis of exopolysaccharide (EPS) [42,43], *tapA* and *sipW* encodes the chaperon and signal peptidase of TasA protein required for amyloid fibre assembly [44], while *bslA* encodes a hydrophobin-like protein [45]. Upregulation of all these biofilm-related genes were observed in the hyphae attached cells that suggests enhanced biofilm matrix production in *B. velezensis* during colonization of the fungal hyphae. Additionally, down regulation of *cwlC* expression indicated, which encodes a cell wall hydrolase [46], is consistent with long chain formation during the onset of biofilm development.

In conclusion, based on the transcriptome analysis we hypothesize the differentiation of *B. velezensis* in cocultures during hyphae colonization, suggesting that interaction with the hyphae of *T. guizhouense* requires the biofilm matrix components of *B. velezensis*.

### 3.4 Hyphae colonization by *B. velezensis* requires the major matrix components

**Fig. 3:**
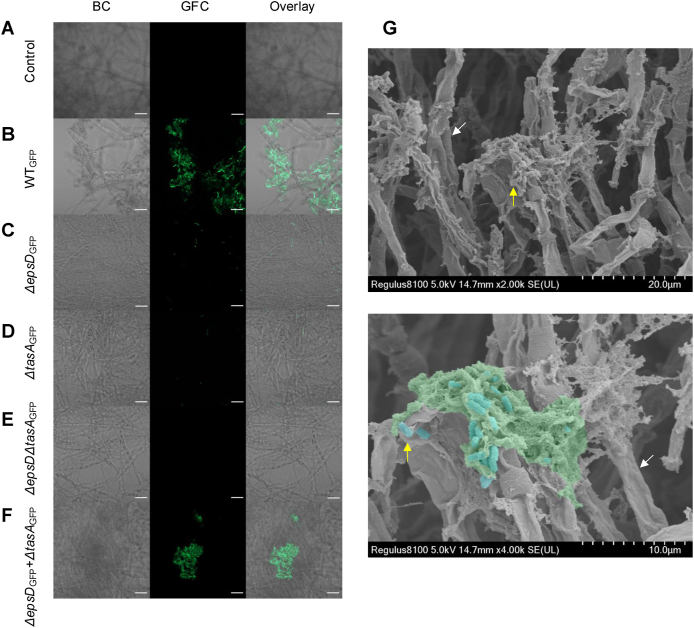
**Attachment of *B. velezensis* on *T. guizhouense* hyphae***.* (A) Control, (B) wild type, (C) Δ*epsD*, (D) Δ*tasA*, (E) Δ*epsD*Δ*tasA*, (F) Δ*epsD +* Δ*tasA*, all images made by CLSM. BC: bright channel; GFC: green fluorescence channel; RFC: red fluorescence channel. Scale bars indicate 20 μm. (G) SEM images of pellets. White arrow: *T. guizhouense*. Yellow arrow and blue color: *B. velezensis*. Green color: EPS of biofilm. Scale bars are depicted in the figures.

To further investigate the role of biofilm matrix production-related genes during hyphae colonization, we removed either one or both genes required for the two major matrix components in the GFP-labeled *B. velezensi*s strain. The wild type, Δ*epsD*, Δ*tasA*, Δ*espD*Δ*tasA*, and mixture of the Δ*epsD* and Δ*tasA* strains were then co-cultured with *T. guizhouense* fungal pellets (Fig. 3A–F). Confocal laser scanning microscopy (CLSM) analysis revealed that the wild-type (WT) *B. velezensis* successfully colonized the hyphae of *T. guizhouense*, whereas none of the mutants were able to colonize the hyphae (Fig. 3A–E & Fig. S4). Furthermore, simultaneous supplementation of both Δ*espD* and Δ*tasA* mutants restored *B. velezensis* biofilm formation on the *T. guizhouense* hyphae, demonstrating the importance of secreted matrix compounds (Fig. 3F). Additionally, scanning electron microscopy (SEM) imaging of the WT-colonized fungal pellet revealed the presence of extracellular polysaccharides and *B. velezensis* cells colonizing the hyphae (Fig. 3G).

## 4 Discussion

BFIs have a profound impact on the nutrition and health of host organisms, as both plants and animals harbor a variety of bacterial and fungal communities [5]. A study on onion tissue bioavailability demonstrated that when inoculated with the mycorrhizal fungus *Golmus intaradices*, *B. subtilis* helper strain significantly increased biomass due to the bacterium's phosphate-solubilizing abilities, leading to nitrogen and phosphorus accumulation in the plant [47].

*T. guizhouense* NJAU 4742 and *B. velezensis* SQR9 have been extensively studied as both have demonstrated positive influence on plant growth [14]. Therefore, a subsequent inoculation of the two PGPM was explored to see the influence of potential BFIs on growth of tomato plants. Importantly, the order of inoculation had no influence on the positive impact of these PGPM on plant yield in soil. While inhibition was observed in the conventional laboratory assays with the two microorganisms inoculated at the same time at distant locations on an agar medium, such competitive interaction was seemingly absent during inoculation of these two strains to the soil, potentially due to the spatial structure created by the soil particles [48] and the plant roots, or due to distinct nutrient availability that lessens the growth rate of these two microorganisms, allowing a more subtle contact between them. Possible explanations include colonization of distinct ecological niches, stress-induced growth promotion, or synergistic metabolic abilities. These possibilities highlight the complexity of rhizosphere interactions and the need for further research to fully understand the BFIs in soil environments.

It is difficult to truly replicate the conditions present in a complex soil environment using a laboratory liquid medium. To circumvent the lack of a reliable system, we customized a coculture method [20,36], in which *T. guizhouense* spores was first allowed to germinate and grow before *B. velezensis* was inoculated. This method provided a stable system to study BFIs. Interestingly, our results indicated *B. velezensis* could colonize on *T. guizhouense* hyphae, contrary to the antagonistic behavior observed when the two microorganisms were inoculated in a distance on PDA medium. Using the coculture system, transcriptome analysis revealed significant differences in gene expression between *B. velezensis* cells colonizing the hyphae versus free living state in the presence or absence of the fungus. Secondary metabolite gene expression was increased in free-living cocultures of *B. velezensis*, suggesting that *B. velezensis* competed for resources, space, and nutrients with *T. guizhouense* by upregulating secondary metabolites [40]. The fungal hyphae colonizing *B. velezensis* cells potentially occupy a distinct ecological niche, due to their reduced mobility and chemotaxis.

As transcriptome analysis of hyphae colonizing bacterial cells suggested an important role biofilm production by *B. velezensis*, mutant strains lacking matrix production were tested and visualize during coculturing using different microscopy methods. As none of the bacterial mutants that lacked the biofilm matrix was able to colonize the fungal hyphae, these experiments clearly demonstrated the essentiality of biofilm matrix production for establishment of BFI under those conditions. The importance of biofilm components in BFIs has been highlighted in previous studies. For example, the biofilm matrix of *B*. *subtilis* is essential for the colonization of the hyphae of the Ascomycota *Aspergillus niger* and the Basidiomycota *Agaricus bisporus* [20].

The outcome of the interaction between bacteria and fungi depends on natural physical interaction [5]. Bacteria coexist within hyphae in various forms, including free-living cells, hyphae attached single cells, endohyphal cells, and matrix embedded cells of biofilms [49]. Previous studies highlighted the role of biofilm matrix during colonization of the hyphae of *Ascomycetes*, *Basidiomycetes* and *Zygomycetes* [50]. However, most of the BFI-related biofilm research focused on mycorrhizal fungi, which inherently cooperate with bacteria. For example, intricate interaction between *B. velenzesis* and the arbuscular mycorrhizal fungi, *Rhizophagus irregularis* suggested that the biosynthesis of specific lipopeptides and antimicrobial compounds in *B. velezensis* is attenuated as a mechanism to ensure stable coexistence of these two microorganisms [51,52].

Future experiments could explore whether in addition to biofilm-mediated colonization, *B. velezensis* can potentially also exploit the *T. guizhouense* hyphae as a “fungal highway”, similar to those observed between *Aspergillus nidulans* and *B. subtilis* [53] or if there is any growth stimulating metabolites are production by *B. velezensis* and *T. guizhouense*. Concurrently, we speculated that within soil ecosystems, a broader spectrum of bacteria interact with hyphae. Identifying and investigating these beneficial interactions among there strains could advance the theoretical understanding of BFIs.

## 5 Conclusion

The interactions within the soil rhizosphere microbiome are complex and their full impact on ecological processes is still being explored. Factors, such as the ecological niche differentiation and variations in growth rates are frequently overlooked. Notably, *Bacillus* and *Trichoderma* exhibit distinct interactions in the laboratory while lack any hindrance on plant growth promotion when applied simultenously. Here, we propose that mitigating the differences in growth rates could offer a simple solution studying BFI in the laboratory. Our findings elucidated that *B. velezensis* could colonize the hyphae of *T. guizhouense* under *in vitro* conditions via biofilm formation with a distinguished transcriptome from non-colonizing bacterial cells. This study provides a novel perspective on BFIs in soil and furnishes a theoretical framework for exploring specific interactions among bacteria, fungi and plants. Such insights are essential for advancing our comprehension of harnessing soil microbiota to improve plant growth and enhance agricultural productivity.

## CRediT authorship contribution statement

**Jiyu Xie:** Writing – original draft, Visualization, Validation, Software, Methodology, Data curation, Conceptualization. **Xinli Sun:** Writing – review & editing, Software, Methodology, Funding acquisition, Conceptualization. **Yanwei Xia:** Writing – review & editing, Software, Methodology. **Lili Tao:** Writing – review & editing, Software, Methodology, Data curation. **Taimeng Tan:** Writing – review & editing, Visualization, Software. **Nan Zhang:** Writing – review & editing, Software, Resources, Conceptualization. **Weibing Xun:** Writing – review & editing, Visualization, Software, Resources, Conceptualization. **Ruifu Zhang:** Writing – review & editing, Supervision, Methodology, Conceptualization. **Ákos T. Kovács:** Writing – review & editing, Supervision, Resources, Methodology, Funding acquisition, Conceptualization. **Zhihui Xu:** Writing – review & editing, Supervision, Resources, Methodology, Funding acquisition, Conceptualization. **Qirong Shen:** Writing – review & editing, Supervision, Conceptualization.

## Declaration of competing interest

The authors declare that they have no known competing financial interests or personal relationships that could have appeared to influence the work reported in this paper.

## Data Availability

Sequencing data have been uploaded to public repository.
