# Supplementary material for: Bridging the Gap: Biofilm-mediated establishment of Bacillus velezensis on Trichoderma guizhouense mycelia
Source: Biofilm. 2024 Nov 16;8:100239. doi: 10.1016/j.bioflm.2024.100239 (PMC11616078; doi:10.1016/j.bioflm.2024.100239)
Supplement: Multimedia component 1 [file mmc1.pdf]

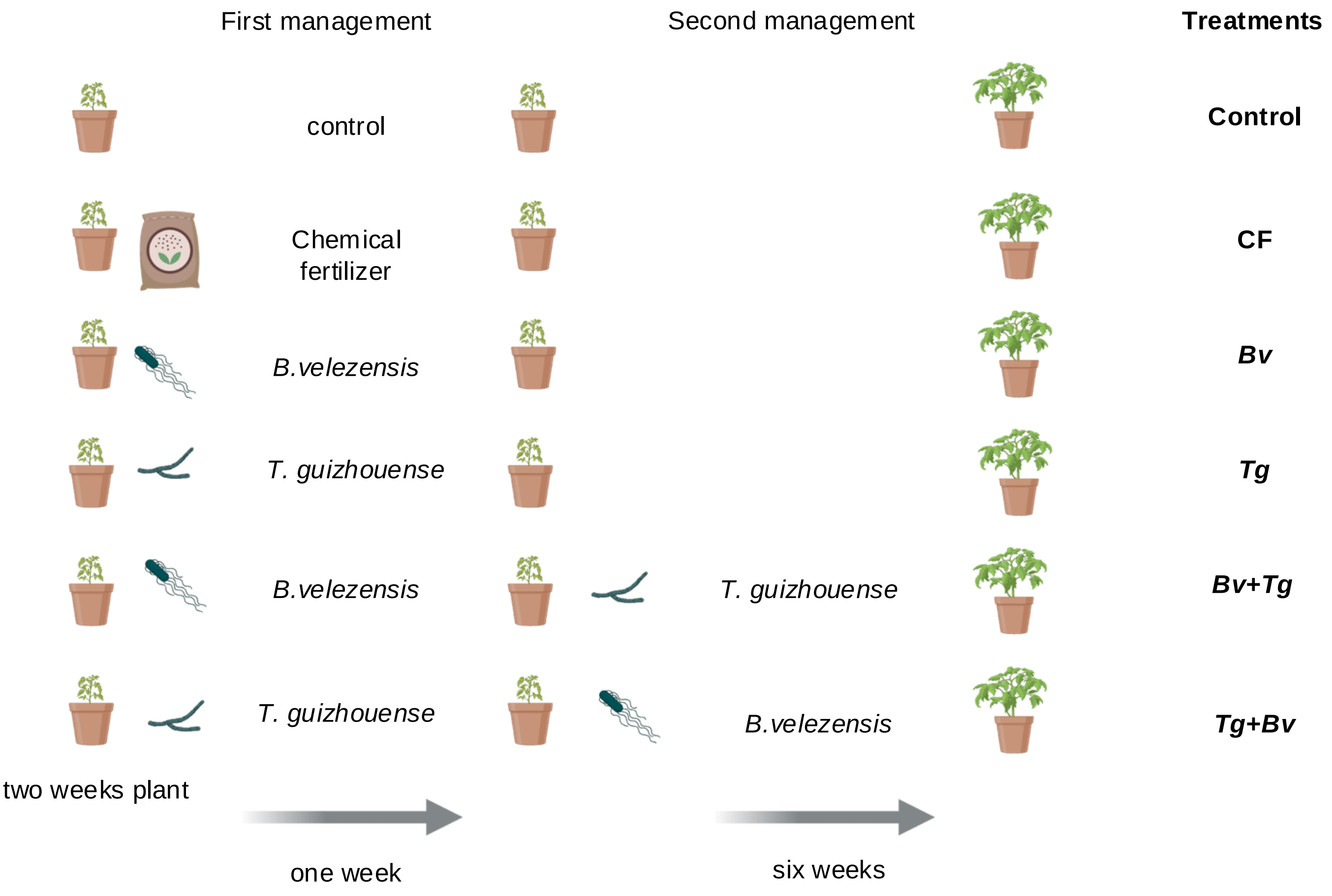

Fig S1 The tomato plant experiments design. Control: water; CF: chemical fertilizer; *Bv*: *B. velezensis*; *Tg*: *T. guizhouense*; *Bv+Tg*: inoculated *B. velezensis* in the soil first then *T. guizhouense*; *Tg+Bv*: inoculated *T. guizhouense* in the soil first then *B. velezensis*.

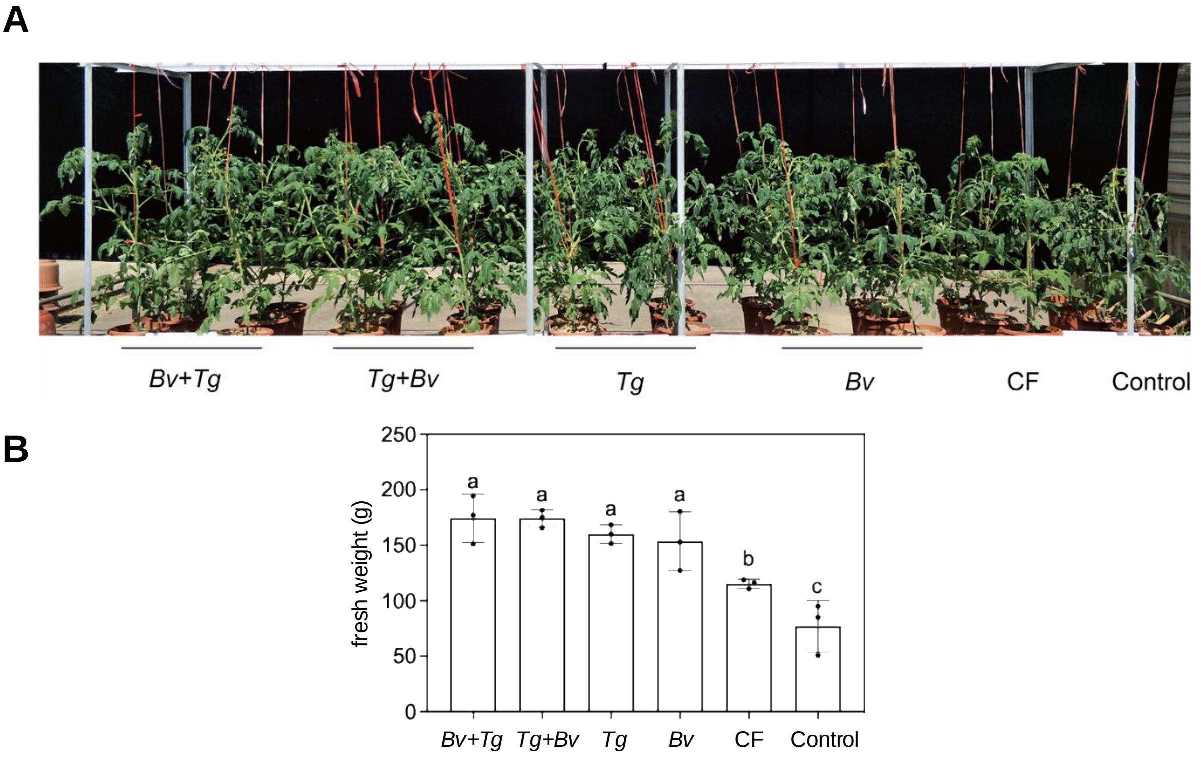

Fig S2 Enhanced growth of tomato under the coculture of *B. velezensis* and *T. guizhouense*. (A) Tomato plants with different treatments. **(B)**. Fresh weight of Plants. Control: water; CF: chemical fertilizer; Bv: *B. velezensis*; Tg: *T. guizhouense*; Bv+Tg: inoculated *B. velezensis* in the soil first then *T. guizhouense*; Tg+Bv: inoculated *T. guizhouense* in the soil first then *B. velezensis*. Bars represent the mean ± s.d. (n=3). Significance test was performed using one-way ANOVA followed by Tukey's multiple comparisons test via Prism 10. Different letters indicate statistically significant ($p < 0.05$) differences.

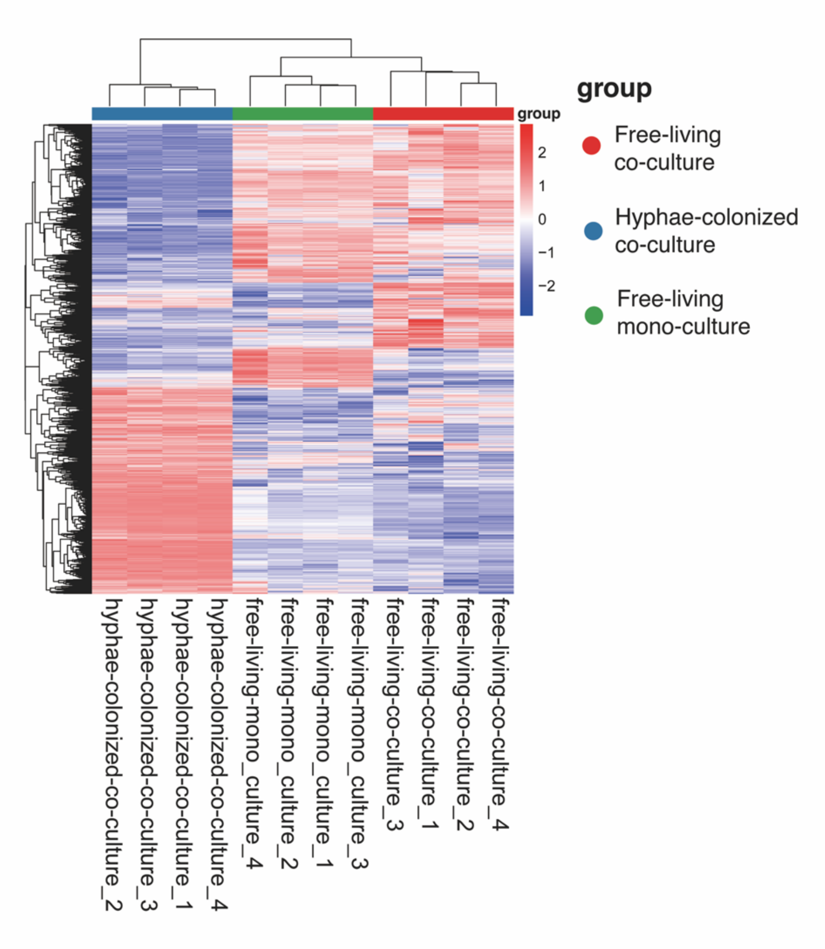

Fig S3 The differential gene expression analysis of *B. velezensis* in different treatments.

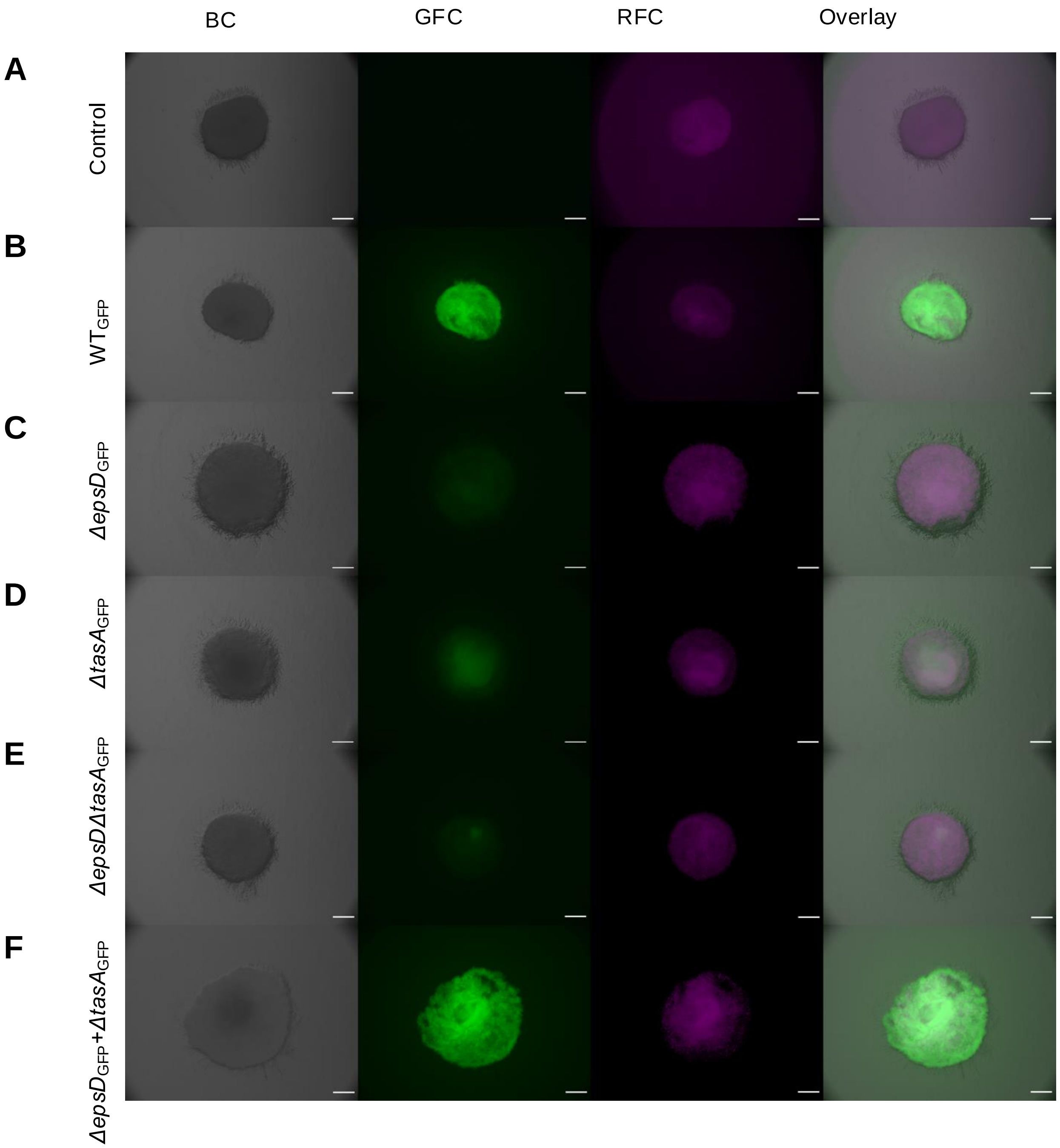

Fig S4 Attachment of *B. velezensis* on *T. guizhouense* hyphae. (A)control, **(B)** wild type, **(C)** *ΔepsD*, **(D)** *ΔtasA*, **(E)** *ΔepsDΔtasA,* **(F)** *ΔepsD* + *ΔtasA*, images made by Stereo Microscopy. BC: bright channel; GFC: green fluorescent channel; RFC: red fluorescent channel. Scale bars indicate 500 μm.
