# Supplementary material for: Bridging the Gap: Biofilm-mediated establishment of Bacillus velezensis on Trichoderma guizhouense mycelia
Source: Biofilm. 2024 Nov 16;8:100239. doi: 10.1016/j.bioflm.2024.100239 (PMC11616078; doi:10.1016/j.bioflm.2024.100239)
Supplement: Multimedia component 2 [file mmc2.pdf]

Table S1: Primers used in this study

Primers	Sequence(From 5' to 3')	Experimental purpose
tasA_UF	TCGCATGCGATGAATCACTTC	To amplify the upstream region of *tasA* gene in *B. velezensis*
tasA_UR	ACTTTTCGGGGAAATGTGCAAGTTCCAAGGCAATGCGA	To amplify the upstream region of *tasA* gene in *B. velezensis*
epsD_UF	CGTCTGCATGCCTGAAAAACG	To amplify the upstream region of *epsD* gene in *B. velezensis*
eps_UR	CGTTACGTTATTAGTTATGAAAAGCTGTACCGCTCCCC	To amplify the upstream region of *epsD* gene in *B. velezensis*
tasA_DF	TATTTAACGGGAGGAAATAATCATTAAATGCGGCCCATGT	To amplify the down stream region of *tasA* gene in *B. velezensis*
tasA_DR	ATCGCTTCTCAGCAGCAGC	To amplify the down stream region of *tasA* gene in *B. velezensis*
epsD_DF	TATAGCATACATTATACGAGATTCATATTGCCGCCTGC	To amplify the downstream region of *epsD* gene in *B. velezensis*
epsD_DR	CATACCGAGCTGTCCGCA	To amplify the downstream region of *epsD* gene in *B. velezensis*
TasA_F	CAGGCTGCTCGATCAGTTTT	To confirm deletion of *tasA* gene in B. velezensis
TasA_R	CGAACGCTGGGATCAAAGTG	To confirm deletion of *tasA* gene in B. velezensis
epsD_F	TAACGGTTCATGACCGCGGG	To confirm deletion of *epsD* gene in B. velezensis
epsD_R	TCGGCAGAAAGCCGGTCA	To confirm deletion of *epsD* gene in B. velezensis
Em_F	TCGCATTGCCTTGGAACTTGCACATTTCCCCGAAAAGT	cloning
Em_R	ACATGGGCCGCATTTAATGATTATTTCCTCCCGTTAAATA	cloning
Spc_F	GGGGAGCGGTACAGCTTTTCATAACTAATAACGTAACG	cloning
Spc_R	GCAGGCGGCAATATGAATCTCGTATAATGTATGCTATA	cloning
